# Flexor Digitorum Brevis Muscle Dry Needling Changes Surface and Plantar Pressures: A Pre-Post Study

**DOI:** 10.3390/life11010048

**Published:** 2021-01-13

**Authors:** Eva María Martínez-Jiménez, Marta Elena Losa-Iglesias, Marta San Antolín-Gil, Daniel López-López, Carlos Romero-Morales, María Benito-de-Pedro, César Calvo-Lobo, Ricardo Becerro-de-Bengoa-Vallejo

**Affiliations:** 1 School of education, Nebrija University, 28015 Madrid, Spain; emartinezji@nebrija.es; 2 Nursing and Stomatology Department, Faculty of Health Sciences, Universidad Rey Juan Carlos, 28922 Madrid, Spain; marta.losa@urjc.es; 3 Villaviciosa de Odón Campus, Universidad Europea de Madrid, 28670 Madrid, Spain; marta.sanantolin@universidadeuropea.es; 4 Health and Podiatry Group, Department of Health Sciences, Faculty of Nursing and Podiatry, Universidade da Coruña, 15403 Ferrol, Spain; daniel.lopez@udc.es; 5 Facultad de enfermería Fisioterapiay Podología, Universidad Complutense de Madrid, 28040 Madrid, Spain; mariabenito1309@gmail.com (M.B.-d.-P.); cescalvo@ucm.es (C.C.-L.); ribebeva@enf.ucm.es (R.B.-d.-B.-V.)

**Keywords:** platform, foot, dry needling, myofascial pain syndrome, trigger point

## Abstract

Background: The effects of the dry needling technique and pain reduction have been demonstrated in numerous quality studies. However, the mechanical effects of dry needling are largely unknown. Methods: A total of 18 subjects with flexor digitorum brevis muscle myofascial trigger point were evaluated pre- and post-deep dry needling. We measured static footprint variables in a pre–post study. Main findings: We found differences in rearfoot maximum pressure (119.22–111.63 KPa; *p* = 0.025), midfoot maximum pressure (13.68–17.26 KPa; *p* = 0.077), midfoot medium pressure (4.75–6.24 KPa; *p* = 0.035) and forefoot surface (86.58–81.75 cm^2^; *p* = 0.020). All variables with significant differences decrease, with the exception of forefoot surface which showed an increase. Conclusions: After flexor digitorum brevis muscle dry needling, midfoot plantar pressures (maximum and medium) and forefoot surface were increased, and rearfoot maximum pressure was decreased.

## 1. Introduction

Myofascial pain syndrome (MPS) generates the most frequent cause of musculoskeletal pain [1]. Myofascial pain is caused by applying pressure to the trigger point, an anatomically hyperirritable localizable structure within the skeletal muscle, which is associated with hypersensitivity symptoms while undergoing clinical exploration via compression, stretch, overload and/or contraction of the muscle [2,3,4]. Because the trigger point alone generates MPS without the need for associated pathologies, this syndrome is considered a specific diagnosis and primary dysfunction [5].

Nociception in MPS is local and referred [4]. Referred pain is maintained and perpetuated by a phenomenon of central sensitization, as it has been observed in clinical trials that anesthetization of the referred pain area reduces pain [6]. Different studies show that central sensitization is a process that can be reversible in myofascial trigger points (MTrPs) with dry needling treatment [7,8]. High-quality studies demonstrate that dry needling can be an effective and safe method for the treatment of myofascial pain [9,10].

Among the effects of the dry needling technique, pain reduction has been demonstrated in numerous quality studies [11,12,13]. However, the mechanical effects of dry needling are largely unknown. It is known that there is a decrease and even elimination of the electromyographic “noise” in the motor endplate after twitch response after this technique is applied [14]. Some authors think that dry needling can modify cholinesterase and acetylcholine receptors, as occurs in muscle regeneration processes [15], or even destroy motor plates [15]. However, biomechanical effects in other studies have shown a greater vertical jump after the application of dry needling compared to a placebo, in addition to a greater range of motion [16,17].

Plantar heel pain is one of the most common musculoskeletal foot-associated pathologies. It is estimated that plantar heel pain will affect 10% of the population at some point in their life [18]. The flexor digitorum brevis trigger point causes referred pain in the heel [4], and is effectively treated by the application of dry needling [19].

The intrinsic muscles of the foot have shown their important involvement in maintaining the stability of the metatarsophalangeal and interphalangeal joints during the take-off phase [20]. Larger intrinsic foot muscle sections are related to higher maximum force, contact area and foot-time integral [21], and the forefoot maximum pressure increment could be explained by the increase in plantar fascia length. A larger plantar fascia and intrinsic foot muscle section are related to higher maximum force [22]. Despite these associations between intrinsic muscle, fascia and footprint status, no investigations have been conducted on the effects of dry needling on any intrinsic muscle and static footprint variables. It is possible that after dry needling, changes in plantar pressures may occur due to the greater elongation capacity of the muscle, as described after therapy [23]. Variations in the surface and pressures of the static footprint are important, as it is known that higher levels of plantar pressures are associated with pathologies such as metatarsalgia [24] and ulcers [25] in a secondary way, so it is necessary to know if adverse events of these types after dry needling are found.

Thus, we did not find any study on the effects of dry needling on intrinsic foot musculature and its effects on plantar pressures in the literature. Therefore, our hypothesis states that after application of dry needling on intrinsic muscles of the foot, the plantar pressures increase in all footprints. The goal of our study was to demonstrate that immediate side effects on plantar pressures with respect to improving the recommendations after treatment with dry needling occur. Currently, only sports rest is recommended after treatment, and there could be own recommendations after applying dry needling to the foot.

## 2. Materials and Methods

### 2.1. Sample Size Calculation

We calculated the sample size with software from Unidad de Epidemiología Clínica y Bioestadística, Complexo Hospitalario Universitario de A Coruña, Universidade A Coruña (www.fisterra.com). In order to observe differences pre- and post-dry needling on plantar pressures, we used a similar study by authors who investigated the acute effects of intermittent stretching on plantar pressures during bipodal standing [26]. These authors found rear foot maximum pressure (KPa) decreased after stretching from 106.24 ± 20.89 to 87.45 ± 22.28 (*p* = 0.004) [23]. Thus, to achieve 95% statistical confidence, an 80% statistical power analysis (α = 0.05, β = 20%) and two-tailed test, a total of 18 participants was required.

### 2.2. Subjects

We selected 18 participants (10 females and eight males) with bilateral active or latent MTrPs in the flexor digitorum brevis muscle. We show socio-demographic characteristics of the sample population in Table 1 [27]. All the participants were informed of the different alternatives to the treatment of their myofascial syndrome in the flexor digitorum brevis, explaining the possible adverse effects of each therapy and benefits. When the participant chose dry needling therapy, they were informed of the possibility of conducting the study. The procedure study and informed consent were explained to them. Once they signed the informed consent, the subjects were included in the study. For subjects who chose another technique or those in whom dry needling therapy was contraindicated, we have not applied it and did not include them.

The inclusion criteria included several parameters: (1) Participants who arrived at the clinic presenting with pain in both heels and who were diagnosed with active or latent MTrP in bilateral flexor digitorum brevis; (2) these specific MTrPs were the only MTrPs diagnosed in the limb or foot; (3) all participants had a normal body mass as obesity can affect plantar pressure distribution [28]; and (4) and had an age range of 27.96 to 36.04 (95% confidence interval [CI]) as body mass could also affect plantar pressures [29].

Exclusion criteria consisted of several parameters: (1) Diagnosis of lower limb injury, such as plantar fasciitis, tendinopathy, bursitis, ligament injuries [27]; (2) a history of previous lower extremity surgery [28]; (3) participants were required not to have undergone ankle stretching or any other treatment [23]; (4) diabetes due to possible elevation of plantar pressure, [30]; (5) deformities of the toes, such as hammer toes and hallux valgus due to their possible alteration in plantar pressure [31]; and (6) receiving anticoagulant therapy [4]. The permission of the required committee was obtained from the Rey Juan Carlos University Ethics Committee with the number 2706201911419. All subjects signed informed consent prior to starting the study. Ethical standards experimentation conformed to the Helsinki Declaration. Public registration of the study was done in clinicaltrials.gov with the number ID: NCT04628312.

### 2.3. Flexor Digitorum Brevis Muscle Dry Needling Therapy

**Figure 1:**
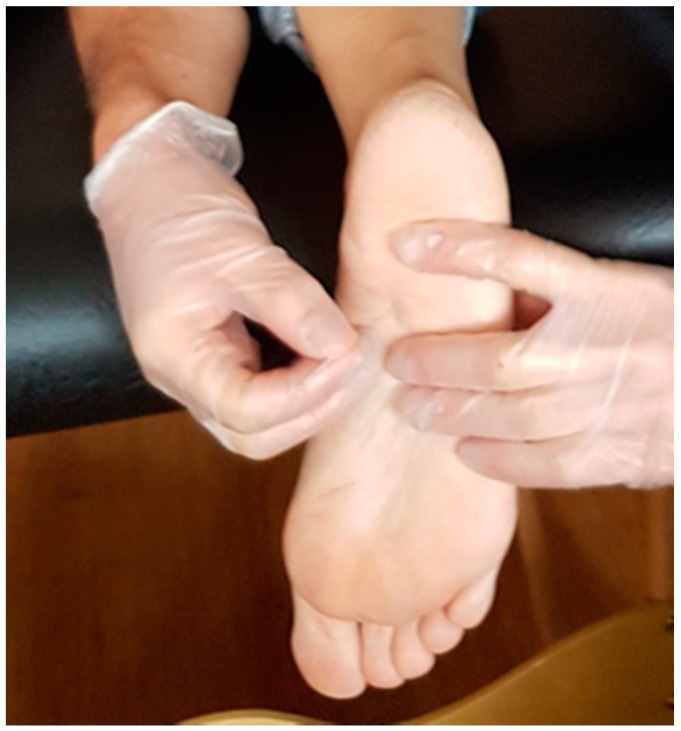
Dry needling therapy of the Flexor digitorum brevis muscle.

The participant’s position was prone, with the foot to be treated off of the table. The clinician placed their arm on the subject’s leg to avoid any withdrawal reaction. First, the location of the trigger point was found using flat palpation. After the required aseptic protocol, gloves and disinfection of the skin was done, the skin was then prepared for the puncture. A dry puncture needle of 0.26 mm × 40 mm (width × length) with a guide (Acimut^®^) is placed over the determined area of greatest pain sensitivity, at which it was estimated that the trigger point is perpendicular to the skin. The needle was pressed down toward the bone, indicating that the fascia and flexor digitorum brevis muscle were reached [11]. Five deviations of the needle of about 30° were made to verify (with the help of the participants) that a local eliciting twitch response and involuntary contraction of the involved muscle occurred, thus verifying that the needle arrived at the trigger point [4,32]. Flexor digitorum brevis muscle dry needling therapy could be seen at Figure 1.

### 2.4. Measurement

The protocol consisted of: (1) diagnosis of MTrP of Flexor digitorum brevis muscle and evaluation of inclusion or exclusion criteria; (2) plantar footprint pre-evaluation in platform [26,29]; (3) deep dry needling of bilateral Flexor digitorum brevis muscle; and (4) immediate post-treatment evaluation. Subjects remained lying down when the puncture was performed, and a local spasm response was observed during the application as a method of verifying the arrival at the trigger point of both muscles [4].

For both pre- and post-evaluation, subjects were instructed to position themselves on the pressure platform with a double limb stance while standing barefoot [33]. The placement of the participant’s feet on the platform was equal distance from the midline [34], and the feet were set at 30° from the midline [35]. During the evaluation, the upper limbs remained hanging loosely along the body [26]. We performed two trials, and the foot area was divided into three bilateral areas: (1) bilateral rear foot; (2) bilateral midfoot; and (3) bilateral forefoot [21,25].

### 2.5. Variables

Ground reaction forces and moments were recorded and digitized with Podoprint, (Medicapteurs; Balma, France). This platform has 2304 sensors at a 400 × 400 mm and a 200 Hz acquisition frequency, and allows the use of an auto-calibrated system at any time.

### 2.6. Statistical Analysis

All data were explored for normality using the Shapiro–Wilk test, as the sample size was <30 subjects [35]. Data were considered normally distributed if *p* > 0.05. Descriptive statistical analysis was performed using mean ± standard deviation (SD) and a 95% CI. For the reliability study, we examined the two types of reliability that exist: (1) relative, which is the degree to which individuals maintain their position or value; and (2) absolute reliability, which is related to the degree of association with different measures of different individuals. We measured absolute reliability with the intra-class correlation coefficient (ICC) and the absolute reliability with standard errors of the mean (SEM), as Bruton, Conway and Holgate [36] and Landis and Koch [37] recommended in their studies and other studies carried out for their reliability analysis [21,26].

Thus, the ICC was used to evaluate the reliability of each parameter to each intra-session trial. To interpret ICC values, we used benchmarks as proposed by Landis and Koch [37]: (1) ≤0.20, slight agreement; (2) 0.21–0.40, fair; (3) 0.41–0.60, moderate; (4) 0.61–0.80, substantial; and (5) ≥0.81, almost perfect.

SEM values were calculated to measure the range of error of each parameter. The SEM was calculated between sessions from the ICCs and SDs. SEM = s_ x.√(1- r_ xx ), in which s_ x was the standard deviation of the observed set of test scores, and r_ xx was the reliability coefficient for these data, which in this case was considered using the ICC.

Two tests of each variable were obtained for each situation (before and after), and the average of two records was used to compared before and after results. The Wilcoxon signed rank test was performed to test for any differences in non-parametric variables, and a paired t-test was used for parametric variables.

Finally, values of normality (VN) of the sample for all variables obtained with the ultrasound and cadaveric dissection were defined. They were obtained from the formula VN = Mean +/−1.96 * SD. From the result of each variable, VN was used to calculate the 95% CI. A *p* value < 0.05 with a CI of 95% was considered statistically significant for all tests (SPSS for Windows, version 26.0; SPSS Inc., Chicago, IL, USA).

The intra-session reliability study of variables and the values of normality in the total population are shown in Table 2.

## 3. Results

The reliability analysis can be seen in Table 2. It can be observed that all of the variables had ≥0.81 as a value. SEM and ICC values confirmed the reliability of the variables.

**Figure 2:**
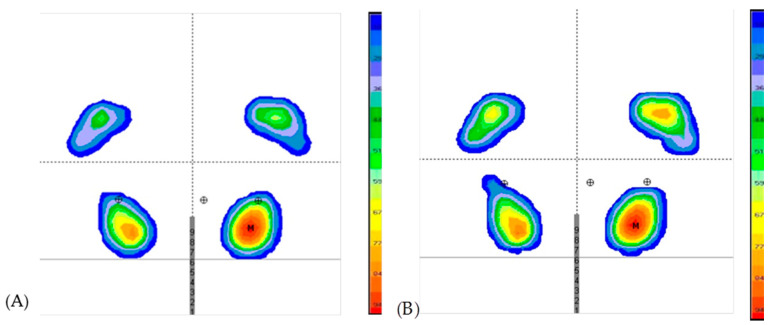
Static pressure distributions of the footprint in a representative subject (**A**) before dry needling. (**B**) after dry needling. Heel part of footprint A and B is located in the lower part. Gray lines and the middle gray numbers indicate the same position in both measures before and after they are invariably applied by the platform. M is the point of highest maximum pressure of the entire static footprint. Rounds with a cross mean are the center of pressures reflected within the tread. The central round is that of both feet, and the lateral ones calculated for each foot. The scale to the right of each footprint is the color representation of the pressure in gram/centimieters^2^ (g/cm^2^), 0 to 29 g/cm^2^ is blue, 29 to 36 g/cm^2^ is purple, 36 to 59 g/cm^2^ is green, 59 at 77 g/cm^2^ is yellow, from 77 to 89 g/cm^2^ is orange, from 89 to 100 g/cm^2^ is red.

Four variables showed a non-normal distribution (*p* < 0.05) in Table 3: (1) midfoot maximum pressure; (2) midfoot medium pressure; (3) midfoot surface; and (4) forefoot medium pressure. Plantar pressures and surface variable values pre- and post-dry needling are also shown in Table 3. After dry needling, midfoot plantar pressures (maximum and medium) and the forefoot surface area increased, and rearfoot maximum pressure decreased. Figure 2 shows the image of the distribution of plantar pressures in a representative subject pre- and post-dry needling.

## 4. Discussion

The results confirm part of our hypothesis in that an increase in the pressures in the midfoot occurred, but we also found unexpected effects on the footprint. After Flexor digitorum brevis dry needling, the medium pressure in the midfoot increases, the surface area in the forefoot increases, and the maximum pressure in the rear foot decreases.

Different investigations of the intrinsic musculature are ongoing. Lee et al. [38] demonstrated that in the intrinsic muscles of the fatigued flat foot, the midfoot and forefoot had an increase in their pressures. In our study, after dry needling of the flexor digitorum brevis, increased midfoot pressures occurred, in addition to when the muscles were fatigued. Farris et al. [20] also demonstrated that in healthy feet, the blockage of intrinsic muscle innervation generates alterations in the stability of the metatarsophalangeal and interphalangeal joints, especially in the take-off phase of walking and running. The authors think that the importance and application of this musculature for the flatfoot is clear. The flatfoot appears to use its intrinsic muscles to try to stabilize the distal joints and the calcaneus, but more research is necessary to verify this finding. Some studies of the increased cross-sectional area of the intrinsic musculature and higher plantar pressure distribution correlate with each other [17]. In the same way, our study demonstrates the importance of the flexor digitorum brevis in maintaining adequate plantar pressure in a static position.

Although after dry needling evidence of decrease in referred pain is found [16], the activation of intrinsic foot muscles may be altered. Studies have found better vertical jumps after thigh dry needling [39], and even the same maximum force after superficial dry needling, in which only the dermis is punctured, and after deep dry needling, reaches the muscle tissue [39]. However, these findings do not fully demonstrate whether this functional improvement after dry needling is due to improvement in reported pain or improvement in contractility and physiological muscle function.

After receiving dry needling treatment for the treatment of their heel pain, the study subjects reported improvement after the application. The clinician’s palpation over the area of the flexor digitorum brevis muscle was soft, indicating relaxation compared to the previous state, meaning it is likely that the cause of the relief of the participant’s pain symptoms was due to the deactivation of the trigger point after application of dry needling. Our results suggest that the authors of this research believe that the influence of pain reduction after puncture is the main component of functional improvement, and that in the case of the present investigation, the loss of function of the flexor digitorum brevis muscle is due to the loss of capacity caused by post-puncture soreness. Post-puncture pain can affect standing in a more decisive way, as part of the area with post-puncture pain is in contact with the ground. We think that the flexor digitorum brevis muscle has a maintenance function of the plantar arch, at least in the group of study subjects, all of them with grade II / III flat feet. When losing some functionality, after dry needling of this muscle, there is a decrease in the internal arch, which secondarily increases the pressures of the midfoot and increases the surface of the forefoot. We have shown that the reduction of pressures in the rear foot is as a consequence of the increase of forefoot surface. In this way, it is reasonable that plantar pressures are related to the morphology of the intrinsic muscles of the foot, as previous research has suggested [22].

We found a decrease in the maximum pressure in the rear foot after application of dry needling. High-heel pressure is associated with greater risk of developing plantar heel pain [40] and is a sign of pathologies, such as Sever’s disease and Type 2 diabetis [41]. We believe that after dry needling, the inhibition of flexor digitorum brevis muscle contributed to the decreases in heel pressure. This decrease appears to support other investigations in which pathologies associated with prolonged standing, which produces an increase in heel pressure [19,42], improve with dry needling treatment [43,44]. Therefore, an indication of the flexor digitorum brevis muscle dry needling could be the occurrence of these pathologies, with an increase in heel pressure such as Sever’s disease, although further research is necessary to understand this association.

Even if it was not an inclusion criteria, all of subjects of the study had type II flatfeet, which could be considered a limitation of this study. The results may not be fully extrapolated to MTrPs in the flexor digitorum brevis muscle in other foot type conditions. Further studies are also necessary to verify the duration of our results and subacute effects. We must emphasize that the results of this study demonstrate the need to make post-foot dry needling recommendations. If subjects with type II flatfeet receive this treatment, they should not walk or play sports, and even avoid standing, as it has been shown that flatfeet already have higher plantar pressures during mid-foot walking that will increase after dry needling [43,44]. The use of orthopaedic insoles and soft-soled shoes, such as sports shoes, which reduce plantar pressures should also be recommended. The importance of the recommendations are considerable, especially if the participant suffers from any neuropathy, diabetes or deformity that implies a major risk of ulceration [43,44], in which non-support of the treated limb after dry needling should be recommended.

## 5. Conclusions

This study demonstrates that after Flexor digitorum brevis dry needling, the medium pressure in the midfoot increases, the surface area in the forefoot increases, and the maximum pressure in the rear foot decreases. Therefore, an indication of the dry needling of the flexor brevis muscle of the fingers could be the appearance of these pathologies with an increase in the pressure of the heel, such as Sever’s disease and Type 2 diabetes. The use of orthopedic insoles and soft-soled shoes, such as sports shoes, should also be recommended in all patients, and even foot support in subjects at risk of ulceration such as diabetics or patients with neuropathy due to increased pressure in the midfoot.

## Figures and Tables

**Table 1:** Socio-demographic characteristics of the sample population.

Variable Total (*n* = 24)	Female Mean ± SD	Female CI 95%	Male Mean ± SD	Male CI 95%
Age (years)	29.60 ± 7.22	(27.96–31.23)	34.37 ± 7.24	(32.73–36.01)
Weight (Kg)	58.60 ± 7.60	(56.87–60.32)	67.50 ± 8.88	(65.48–69.51)
Height (cm)	161.00 ± 7.64	(159.26–162.73)	171.87 ± 1.35	(171.56–172.18)
BMI (Kg/m^2^)	22.78 ± 3.42	(22.00–23.55)	22.73 ± 2.95	(22.06–23.40)
Size of shoe	38.00 ± 1.56	(37.64–38.35)	40.87 ± 1.36	(40.56–41.18)

Abbreviation: Kg (Kilograms); cm (centimeters), BMI (Body Mass Index), SD (standard deviation), CI 95% (confidence interval 95%).

**Table 2:** Analysis of Intrasession Reliability of the Variables Studied and Values of Normality in Total Population.

	Pre-Test (*n* = 18)			Post-Test (*n* = 18)		
Variable	ICC (95% CI)	SEM	Values of Normality 95% CI	ICC (95% CI)	SEM	Values of Normality 95% CI
Rearfoot maximum pressure (kPa)	0.912 (0.761–0.967)	5.99	2483.56–2565.95	0.849 (0.598–0.943)	6.84	1925.33–2001.45
Rearfoot medium pressure (kPa)	0.851 (0.611–0.944)	1.06	243.77–257.41	0.946 (0.856–0.980)	0.96	231.00–253.97
Rearfoot surface (cm^2^)	0.966 (0.911–0.987)	2.23	1040.37–1087.84	0.925 (0.804–0.972)	3.05	943.44–987.11
Midfoot maximum pressure (kPa)	0.985 (0.960–0.994)	1.99	190.68–254.46	0.953 (0.878–0.982)	3.70	261.17–328.08
Midfoot medium pressure (kPa)	0.999 (0.997–1.00)	0.22	19.66–428.46	0.868 (0.643–0.952)	3.13	36.97–70.84
Midfoot surface (cm^2^)	0.996 (0.990–0.999)	1.46	337.67–428.46	0.955 (0.880–0.983)	4.59	317.39–402.38
Forefoot maximum pressure (kPa)	0.848 (0.583–0.944)	4.53	806.42–852.01	0.975 (0.933–0.991)	1.46	664.51–700.93
Forefoot medium pressure (kPa)	0.977 (0.939–0.991)	1.41	202.92–239.38	0.962 (0.897–0.986)	0.65	84.83–98.08
Forefoot surface(cm^2^)	0.873 (0.658–0.953)	3.41	1904.79–1993.03	0.976 (0.936–0.991)	3.41	1998.21–2084.73

Abbreviation: Kg (Kilograms); cm (centimeters), cm^2^ (centimeters^2^), SD (standard deviation), CI 95% (confidence interval 95%).

**Table 3:** Stabilometry and static footprints variables before and after dry needling.

	Pretest (*n* = 18)		Posttest (*n* = 18)		
Variable	Mean ± SD (CI 95%)	Median (RI)	Mean ± SD (CI 95%)	Median (RI)	*p*
Rearfoot maximum pressure (kPa)	119.22 ± 21.18 (114.43–124.02)	121.70 (24.41)	111.63 ± 17.62 (107.64–115.62)	111.72 (24.78)	0.025 ^b,^*
Rearfoot medium pressure (kPa)	42.73 ± 5.76 (41.42–44.04)	42.15 (4.70)	41.38 ± 5.86 (40.05–42.71)	43.07 (6.26)	0.231 ^b^
Rearfoot surface (cm^2^)	87.87 ± 12.11 (85.13–90.61)	89.25 (16.50)	86.65 ± 11.14 (84.12–89.17)	88.50 (18.8)	0.198 ^b^
Midfoot maximum pressure (kPa)	13.68 ± 16.27 (10.00–17.37)	5.25 (32.95)	17.26 ± 17.07 (13.25–21.27)	10.20 (38,27)	0.077 ^a^
Midfoot medium pressure (kPa)	4.75 ± 7.05 (3.15–6.34)	0.00 (14.27)	6.24 ± 8.64 (4.28–8.19)	0.00 (14.15)	0.035 ^a,^*
Midfoot surface (cm^2^)	16.54 ± 23.16 (11.29–21.79)	1.75 (43.8)	16.6 ± 21.68 (11.69–21.50)	4.75 (39.1)	0.916 ^a^
Forefoot maximum pressure (kPa)	71.30 ± 11.63 (68.66–79.93)	71.45 (8.63)	73.49 ± 9.29 (71.39–75.60)	74.62 (14.46)	0.184 ^b^
Forefoot medium pressure (kPa)	23.78 ± 9.30 (21.68–25.89)	25.77 (5.15)	27.06 ± 3.38 (26.30–27.83)	26.45 (4.15)	0.139 ^a^
Forefoot surface(cm^2^)	86.58 ± 22.51 (81.48–91.68)	81.50 (35.4)	92.50 ± 22.07 (87.50–97.50)	81.75 (31.3)	0.020 ^b,^*

Abbreviations: SD, Standard Deviation; CI 95%, Confidence interval 95%; RI, Range interquartile; KPa, Kilopascals; ^a^ *p* value in from Wilcoxon Signed-Rank Test; ^b^ *p* value from paired t-test; A *p* value < 0.05 with a confidence interval of 95% was considered statistically significant, * statistical significance.

## Data Availability

The data will be available under request.
